# DNA as a Phosphate Storage Polymer and the Alternative Advantages of Polyploidy for Growth or Survival

**DOI:** 10.1371/journal.pone.0094819

**Published:** 2014-04-14

**Authors:** Karolin Zerulla, Scott Chimileski, Daniela Näther, Uri Gophna, R. Thane Papke, Jörg Soppa

**Affiliations:** 1 Institute for Molecular Biosciences, Biocentre, Goethe-University, Frankfurt, Germany; 2 Department of Molecular and Cell Biology, University of Connecticut, Storrs, Connecticut, United States of America; 3 Department of Molecular Microbiology and Biotechnology, George S. Wise Faculty of Life Sciences, Tel Aviv University, Ramat Aviv, Tel Aviv, Israel; National Cancer Institute, United States of America

## Abstract

*Haloferax volcanii* uses extracellular DNA as a source for carbon, nitrogen, and phosphorous. However, it can also grow to a limited extend in the absence of added phosphorous, indicating that it contains an intracellular phosphate storage molecule. As *Hfx. volcanii* is polyploid, it was investigated whether DNA might be used as storage polymer, in addition to its role as genetic material. It could be verified that during phosphate starvation cells multiply by distributing as well as by degrading their chromosomes. In contrast, the number of ribosomes stayed constant, revealing that ribosomes are distributed to descendant cells, but not degraded. These results suggest that the phosphate of phosphate-containing biomolecules (other than DNA and RNA) originates from that stored in DNA, not in rRNA. Adding phosphate to chromosome depleted cells rapidly restores polyploidy. Quantification of desiccation survival of cells with different ploidy levels showed that under phosphate starvation *Hfx. volcanii* diminishes genetic advantages of polyploidy in favor of cell multiplication. The consequences of the usage of genomic DNA as phosphate storage polymer are discussed as well as the hypothesis that DNA might have initially evolved in evolution as a storage polymer, and the various genetic benefits evolved later.

## Introduction

The advantages of polyploidy that led to its development in evolution has long been discussed in the framework of eukaryotes, because prokaryotes were long thought to be typically monoploid (a single copy of the chromosome before replication), which is often erroneously termed “haploid”. Evolutionary explanations for organisms with homologous sets of chromosomes have long been linked to the invention of sexual reproduction [1], and have been developed from mathematical modeling using population genetics principles and assumptions. Those analyses indicate that ploidy levels ≥2 n could be selectively advantageous by preventing the expression of deleterious recessive alleles [2]. Additional hypotheses are interconnected with high recombination rates [2] or cell size and r vs. K selection [3]. However, in recent years polyploidy has been demonstrated to be widespread in bacteria and archaea as well [4]–[7], indicating that it is an ancient trait preceding eukaryotes, and that any explanation for the origin and maintenance of higher ploidy levels must address asexually reproducing prokaryotes.

A few polyploid prokaryotic species and their probable selective advantage of polyploidy have been well characterized. For example, cells from the unusually large bacterium *Epulospicium* type B, whose dimensions make it visible to the naked eye, are estimated to contain 50,000–120,000 chromosome copies per cell, which are positively correlated with cytoplasmic volume [8]. Because of diffusion limitations, the extreme polyploidy of *Epulospicium* is thought to be necessary for efficient gene expression. Though interesting and biological relevant, this polyploidy system probably has evolved rather late in evolution because a giant cell size requires a cytoskeleton and advanced intracellular transport. Another example is the bacterium *Deinococcus radiodurans*, which survives high doses of ionizing radiation that generate hundreds of double strand breaks. Its survival strategy relies on polyploidy for performing interchromosomal recombination, which is necessary for repairing its fragmented DNA [9], [10]. While X-ray irradiation is used to induce double strand breaks in the laboratory, the cause of double strand breaks and chromosome fragmentation in nature is desiccation. Polyploidy as a basis for the repair of scattered chromosomes probably evolved early, nevertheless, it requires the pre-existence of a sophisticated DNA repair system. In summary, nearly 10 putative evolutionary advantages that led to the development of polyploidy at different times in different prokaryotic lineages have been discussed [4]–[7], most of which require the pre-existence of homologous recombination. Here we add an additional evolutionary advantage of polyploidy that does not require the pre-existence of homologous recombination, namely the usage of genomic DNA as a storage polymer. The experiments revealing that a prokaryotic species uses DNA as a storage polymer were performed with *Haloferax volcanii*, a halophilic archaeon.

Halophilic Archaea of the family *Halobacteriaceae* are polyploids with phenotypic traits consistent with polyploidy. Both species *Hfx. volcanii* and *Halobacterium salinarum* are demonstrated to contain more than 20 chromosome copies during exponential phase and 10 during stationary phase [4]. *Hbt. salinarum* has been shown to be very resistant to gamma radiation [11], and *Halorubrum chaoviator* strain Halo-G survived the conditions of outer space for two weeks [12], which would be unlikely if these species were monoploids. Furthermore, halobacteria in general experience homologous recombination and gene transfer from distant species [13], [14] and *Halorubrum* populations exist in genetic equilibrium [15]. Haloarchaea produce heterozygous cells after fusion of membranes and cell walls [16]. This is even true for different species thus displaying an unusually low species barrier to homologous recombination [17] and thus can account for their genetic exchange partner promiscuity. These phenotypic characteristics of haloarchaea show that they make intensive use of various genetic advantages of polyploidy. However, here we show that nutrient availability determines ploidy level and that extracellular and intracellular genomic DNA is used as a storage polymer. Notably, it is also shown that *Hfx. volcanii* diminishes genetic advantages of polyploidy under conditions of phosphate starvation.

## Results

### Intracellular storage capacities and growth on external genomic DNA

**Figure 1:**
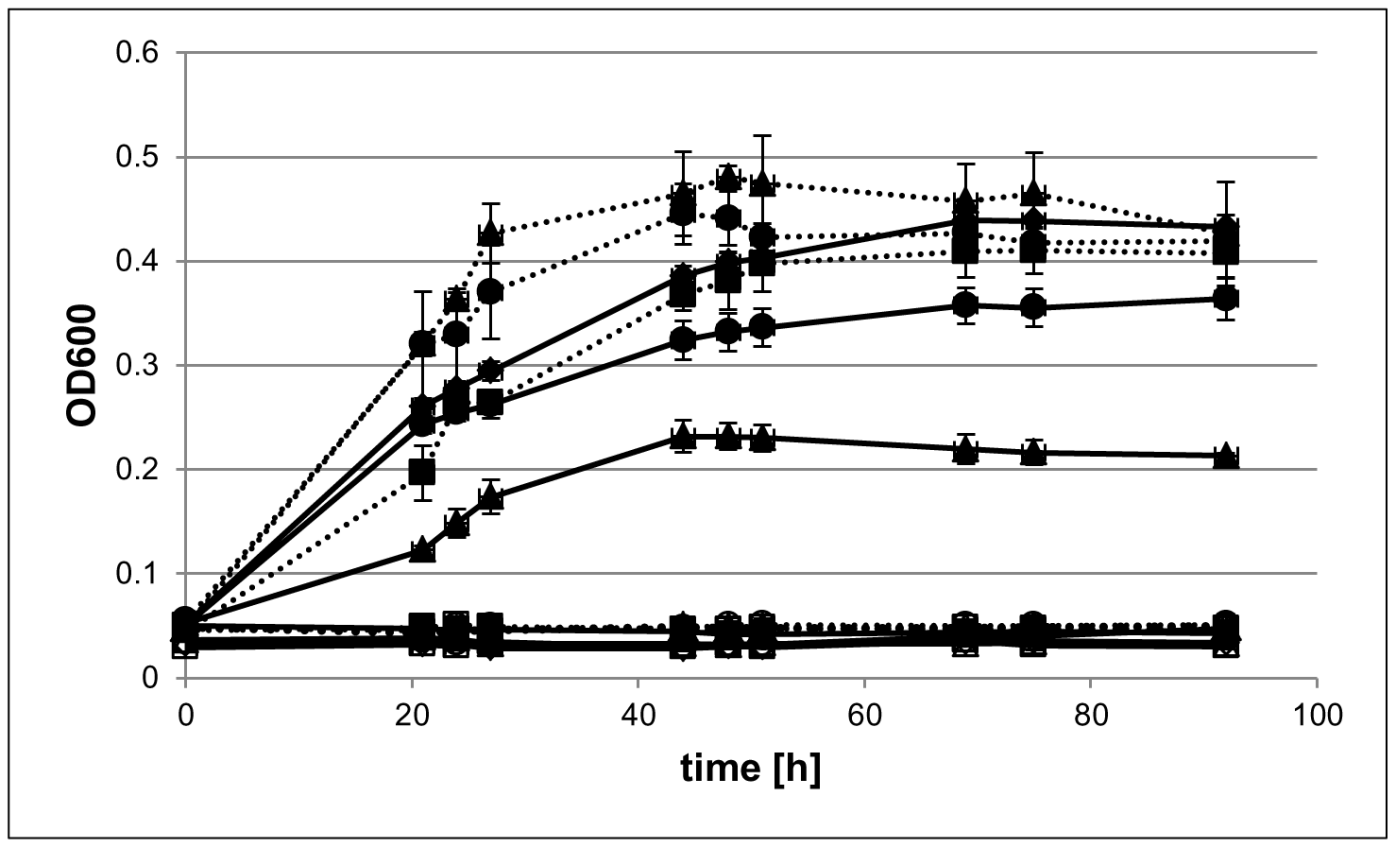
*Hfx. volcanii* uses external DNA as a nutrient source and contains internal P and N storages. *Hfx. volcanii* was grown in microtiter plates in synthetic medium with added carbon (C), nitrogen (N), and phosphate (P) as positive control (diaments). In additional cultures each one of the three nutrients was replaced with genomic DNA (dotted lines), i.e. C was replaced (squares), N was replaced (circles), and P was replaced (triangles). In further cultures each one of the respective nutrients was omitted without replacement (solid lines), i.e. C was omitted (squares), N was omitted (circle), and P was omitted (triangles). To verify that spill over did not occur, for each medium also non-inoculated controls (sterile controls) were performed (open symbols). In each case average values of three independent cultures and their standard deviations are shown.

The first aim of this study was to clarify whether *Hfx. volcanii* can use external (environmental) genomic DNA as a source of carbon (C), nitrogen (N), and/or phosphorous (P). Control cultures supplemented with all three nutrients in the form of glucose, ammonium chloride and potassium phosphate were compared to cultures in which each one of the substances, respectively, was omitted. In each case three independent cultures were grown, and average growth curves and their standard deviations are shown in Fig. 1. In the absence of externally added genomic DNA no growth occurred when C was omitted, indicating that *Hfx. volcanii* has no intracellular carbon storage (Fig. 1, curve –C). In contrast, considerable growth occurred when P was omitted, showing that *Hfx. volcanii* contains an intracellular phosphate storage pool. The growth yield was about 40% of the control culture grown in the presence of all three nutrients. Also the omission of ammonium chloride resulted in considerable growth with a growth yield of about 80% of the control culture. However, in preparation of future genetic experiments *Hfx. volcanii* strain H26 was used, which is auxotrophic for uracil. Therefore, uracil had to be supplemented, which might have been used as nitrogen source, and thus the experiment is uninformative about the absence or presence of an internal nitrogen storage pool.

The addition of external genomic DNA to cultures lacking any of the three nutrients in all three cases enhanced the growth yield, revealing that genomic DNA can be a source for C, N, and P for *Hfx. volcanii* (filled symbols and dotted lines in Fig. 1). The addition of genomic DNA to cultures lacking phosphate or ammonium resulted even in faster growth compared to the control culture grown in the presence of all three nutrients. Only the culture with genomic DNA instead of glucose as a C-source had a substantially lower growth rate, showing that genomic DNA is metabolized more slowly than glucose as a carbon source.

These results revealed on the one hand that external (environmental) DNA can be used as a source for C, N, and P, and on the other hand showed that *Hfx. volcanii* must have intracellular storage capacities for P, but not for C. In the following experiments we concentrated on the usage of external genomic DNA as a source of P and the identity of the intracellular P storage polymer.

**Figure 2:**
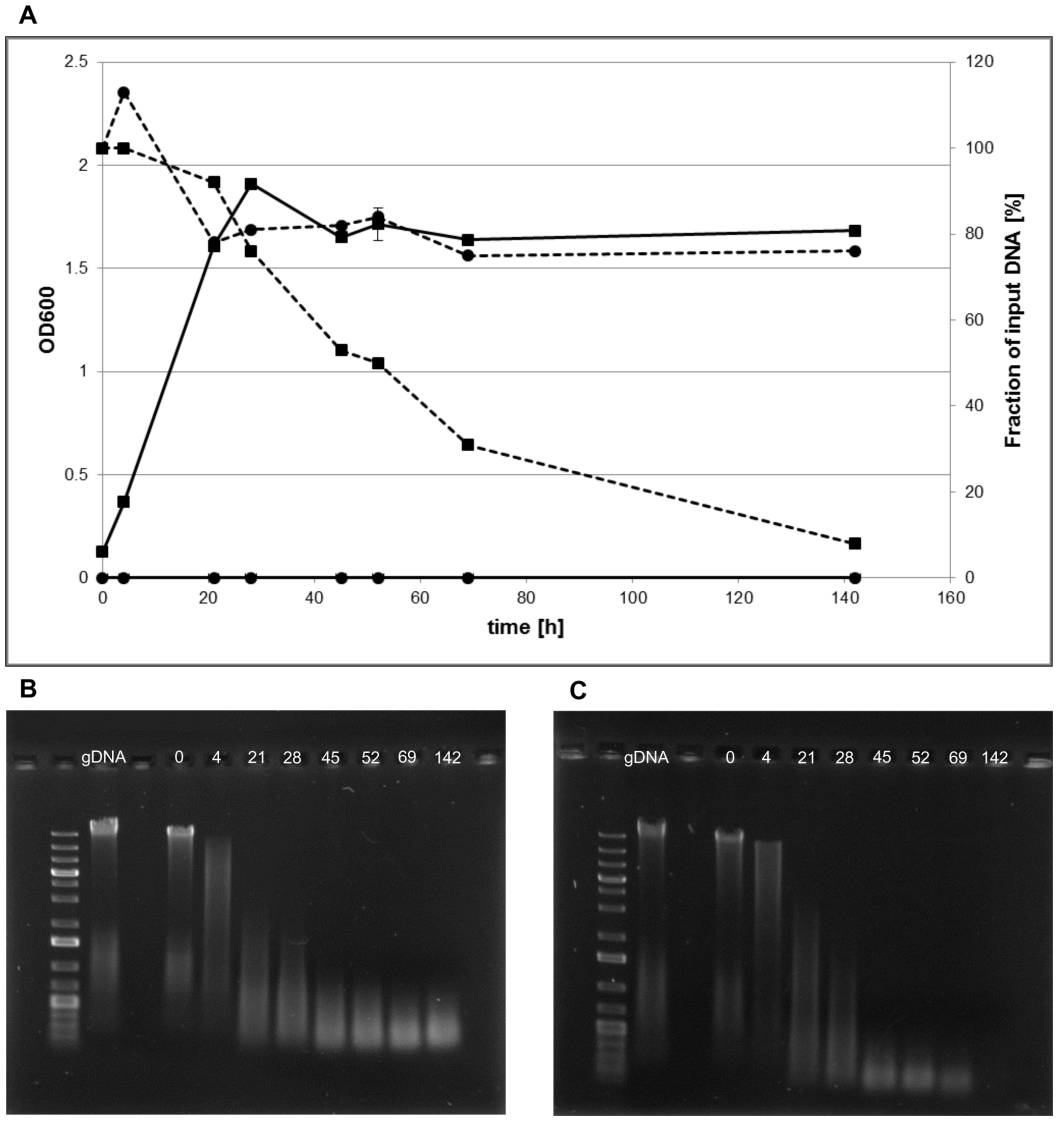
*Hfx. volcanii* consumes high molecular weight chromosomal DNA. Three *Hfx. volcanii* cultures were grown in synthetic medium with chromosomal DNA as sole source of phosphorous and a growth curve was recorded (solid line, squares). As negative controls three non-inoculated cultures were incubated under identical conditions (solid line, circles). At the indicated times the optical densities were recorded and aliquots were removed for the quantification of the DNA content. Average optical densities and their standard deviations are shown (solid lines). The cells were pelleted by centrifugation and the DNA content of the supernatants was analyzed by analytical agarose gel electrophoresis (compare B and C). The DNA concentration was quantified using ImageJ, and average values and their standard deviations are shown (dotted lines, circles for the mock-treated non-inoculated control, squares for the inoculated culture). **B**. The supernatants of the aliquots of non-inoculated negative control cultures were dialyzed to remove salts and analyzed by analytical agarose gel electrophoresis. One representative gel is shown. For comparison the input DNA (gDNA) and a size marker (1 kb plus) were included. **C**. The supernatants of the aliquots of cultures grown with genomic DNA as phosphate source were dialyzed to remove salts and analyzed by analytical agarose gel electrophoresis. One representative gel is shown. For comparison the input DNA (gDNA) and a size marker (1 kb plus) were included.

To further confirm that high molecular weight genomic DNA was indeed the source of the phosphorous, and not potential impurities or contaminations, *Hfx. volcanii* was grown in the presence of DNA and the absence of any other supplemented P. As a control, non-inoculated cultures were incubated under identical conditions. Fig. 2A shows average OD_600_ values of three independent cultures and their standard deviations. Notably, the OD_600_ values of Fig. 2A cannot be compared to that of Fig. 1, because in this experiment cultures were grown in Erlenmeyer flasks and not in microtiter plates and thus path length, photometer, and +/−dilution prior to measurements differ in the two experiments. At the eight time points indicated in Fig. 2 aliquots were removed and the cells were pelleted by centrifugation. The DNA content of the supernatant was analyzed by analytical agarose gel electrophoresis (after dialysis to remove the high salt concentration of the medium). Fig. 2B shows one representative gel of the mock treated culture. It can be seen that the high molecular weight input genomic DNA is broken into small fragments, either by chemical hydrolysis or, more probable, by mechanical shearing forces due to the shaking with 250 rpm. The amount of DNA was quantified using the program ImageJ and the result is included in Fig. 2A (filled squares, dotted line). Within 21 hours the values dropped to 80% and then stayed constant throughout the remaining 120 hours of the experiment. Most probably the initial drop of 20% in integrated signal intensity is not due to a real loss of DNA, but to a broader distribution of the fragments in the gel compared to the full-size genomic DNA. Fig. 2C shows one representative gel of the DNA content in the supernatants of the inoculated culture. In contrast to the mock treated culture the amount of DNA steadily decreased and less than 10% of the input DNA was left after 142 hours. Taken together, these results clearly show that *Hfx. volcanii* can use external (environmental) genomic DNA as a source of phosphorous (Fig. 1 and 2) and also as a source of carbon and nitrogen (Fig. 1).

### Genomic DNA is the intracellular storage polymer of phosphate

**Figure 3:**
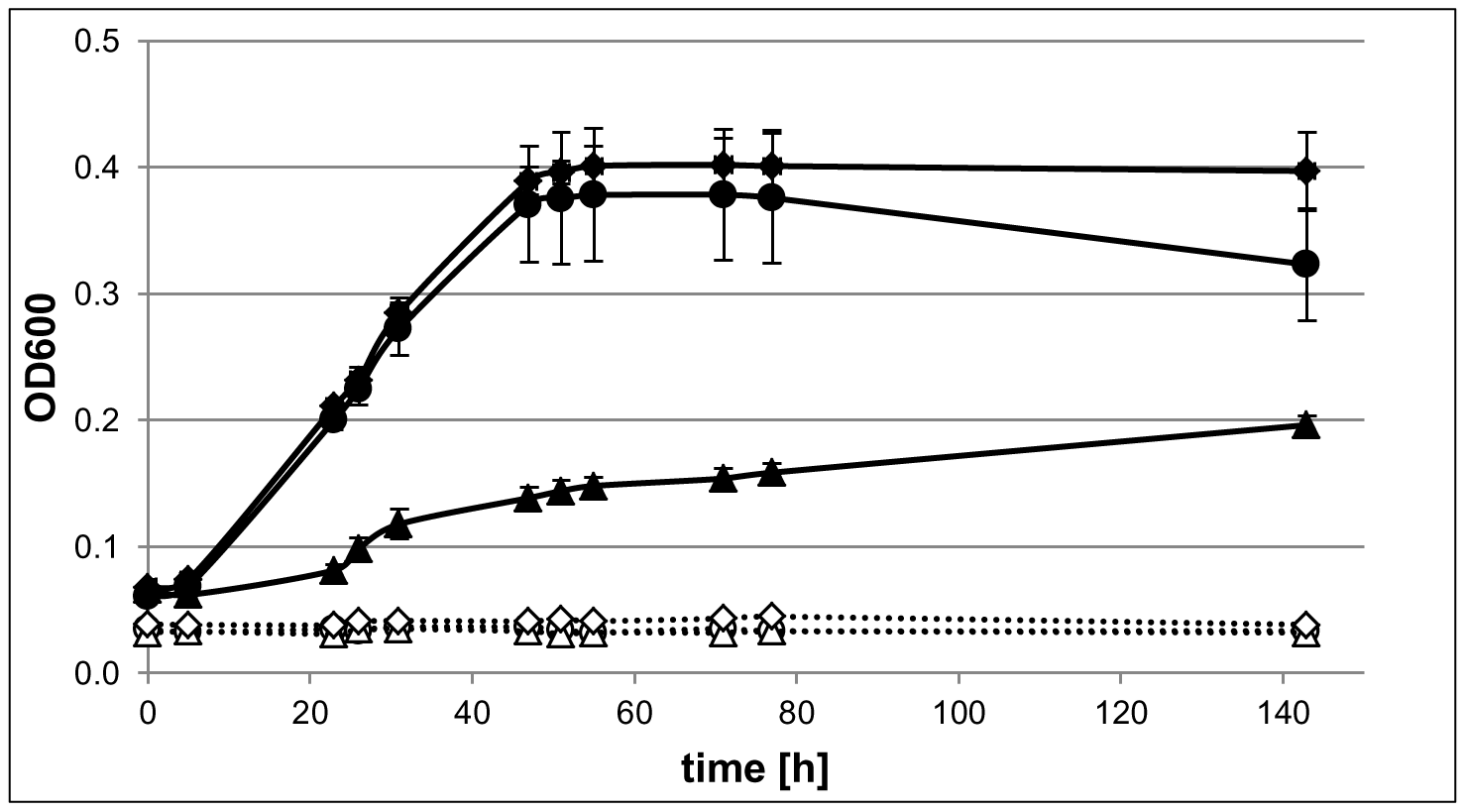
Comparison of growth with different phosphate concentrations and with DNA. *Hfx. volcanii* was grown in microtiter plates in synthetic medium in the absence of any added phosphate source (triangles) and in the presence of, respectively, 1 mM (standard concentration, solid circles) and 10 mM phosphate (diamonds). Non-inoculated sterile controls were also incubated (dotted lines). Growth was followed by measuring the optical density at 600 nm. Average values of three independent cultures and their standard deviations are shown.

For a further characterization of the growth of *Hfx. volcanii* in the absence of any externally added P source cultures were grown in the presence of two different phosphate concentrations (1 mM, and 10 mM) and in the absence of added P. The results are shown in Fig. 3. Growth with 1 mM and with 10 mM phosphate was identical, indicating that phosphate is not the limiting nutrient under these conditions. Again, considerable growth was observed in the absence of added P, indicating that the liberation of phosphate from the intracellular phosphate storage polymer is growth rate-limiting. The OD_600_ at the start of the experiment was about 0.05. The sterile controls showed that the microtiter plates had and OD_600_ of about 0.03 and thus the inoculum had an OD_600_ of about 0.02. After 140 h growth in the absence of added P the cells had an OD_600_ of about 0.17 (measured OD_600_ of 0.2 minus the OD_600_ of the sterile control, 0.03). This is an 8.5-fold increase in OD_600_, which would be equivalent to about three doublings in the absence of added phosphate if the light scatter of the cells would not change. Microscopic observation of the cells indicated that they had normal morphology and were of similar size.

**Figure 4:**
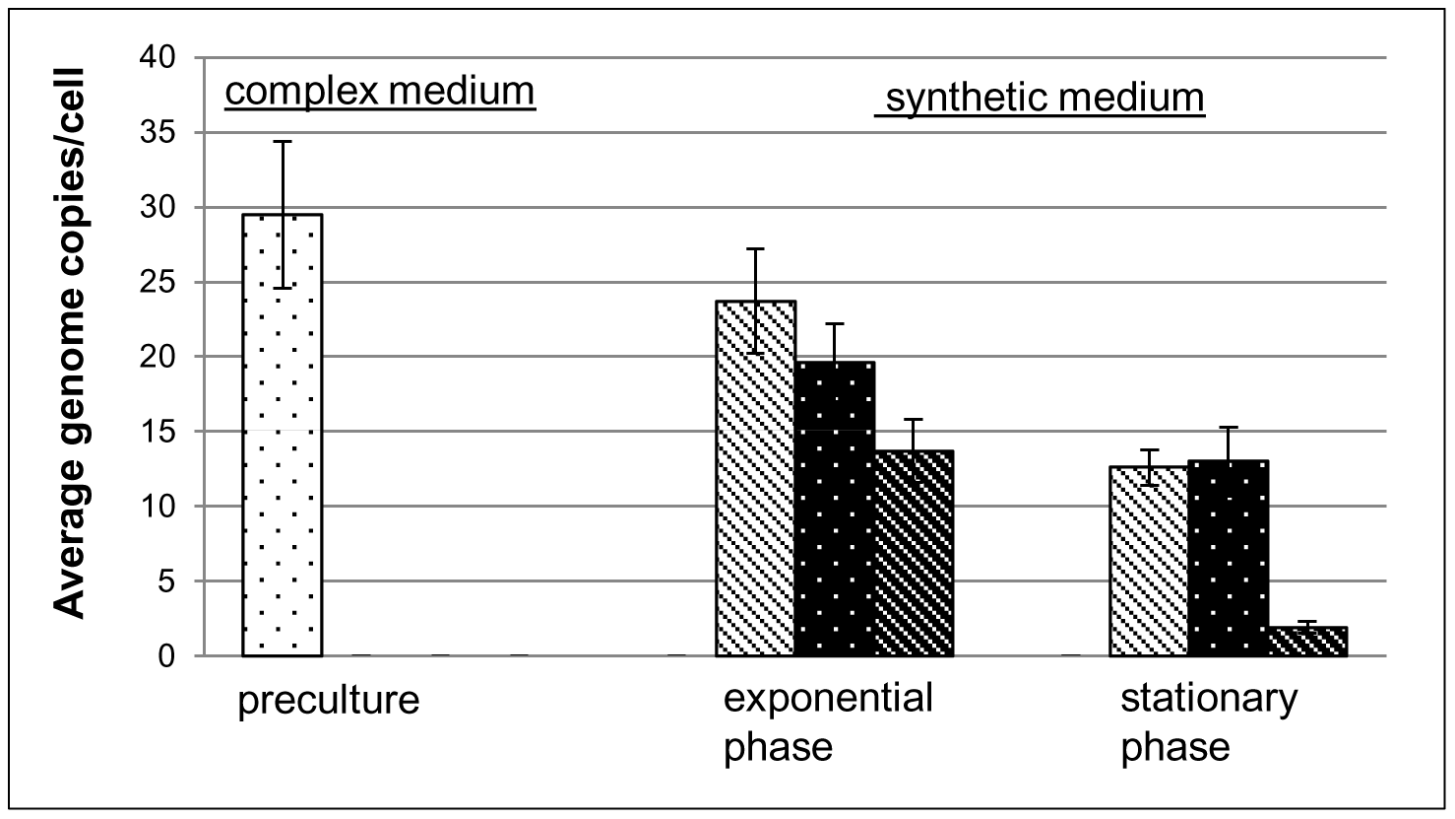
Chromosomal copy numbers during and after growth with and without added phosphate. *Hfx. volcanii* was grown in synthetic medium in the presence of 10 mM and 1 mM phosphate and in the absence of phosphate, respectively. Aliquots were removed during mid-exponential growth phase and at stationary phase (compare text). An aliquot from the pre-culture used for inoculation was also included. Cells were harvested by centrifugation and the chromosome copy number was quantified using Real Time PCR. Three biological replicates were performed and average values and standard deviations are shown, from left to right 10 mM phosphate, 1 mM phosphate, and no externally added phosphate.

These observations that *Hfx. volcanii* maintains an intracellular phosphorous storage and our previous results that *Hfx. volcanii* is highly polyploid and contains about 25–30 copies of the chromosome in exponential phase [4] led to the hypothesis that genomic DNA might be the intracellular phosphate storage polymer. To test our hypothesis we used *Hfx. volcanii* cells grown to exponential phase in complex medium as an inoculum for assessing growth in synthetic media supplemented with two different phosphate concentrations (1 mM, and 10 mM) and no added phosphate. Using quantitative PCR (qPCR), chromosome copy numbers were estimated for the inoculum (an estimate of the pre-growth condition) as well as cells grown to exponential phase and stationary phase without added phosphate (exponential phase: 9.4×10^7^ cells/ml, stationary phase: 2.7×10^8^ cells/ml) and with 1 mM and 10 mM phosphate supplementation (for both: exponential phase: 5.2×10^8^ cells/ml, stationary phase: 1.3×10^9^ cells/ml). During exponential growth, the phosphate concentration was found to influence the ploidy level, with 24 copies on average in cells grown with 10 mM phosphate, 19 copies in cells grown with 1 mM phosphate, and 14 in cells grown in the absence of an added source of phosphorous (Fig. 4). Stationary phase cells that were grown in the presence of added phosphate (10 and 1 mM) maintained approximately 13 chromosomal copies of their genome. However, in the absence of phosphate supplementation, stationary cells had on average reduced their genome copy number to two. This result showed that *Hfx. volcanii* indeed uses genomic DNA as a phosphate storage polymer and indicated that it diminishes the putative genetic and long-term advantages of polyploidy (e.g. DNA repair, desiccation resistance, long term survival) to enable short-term reproductive gains.

**Figure 5:**
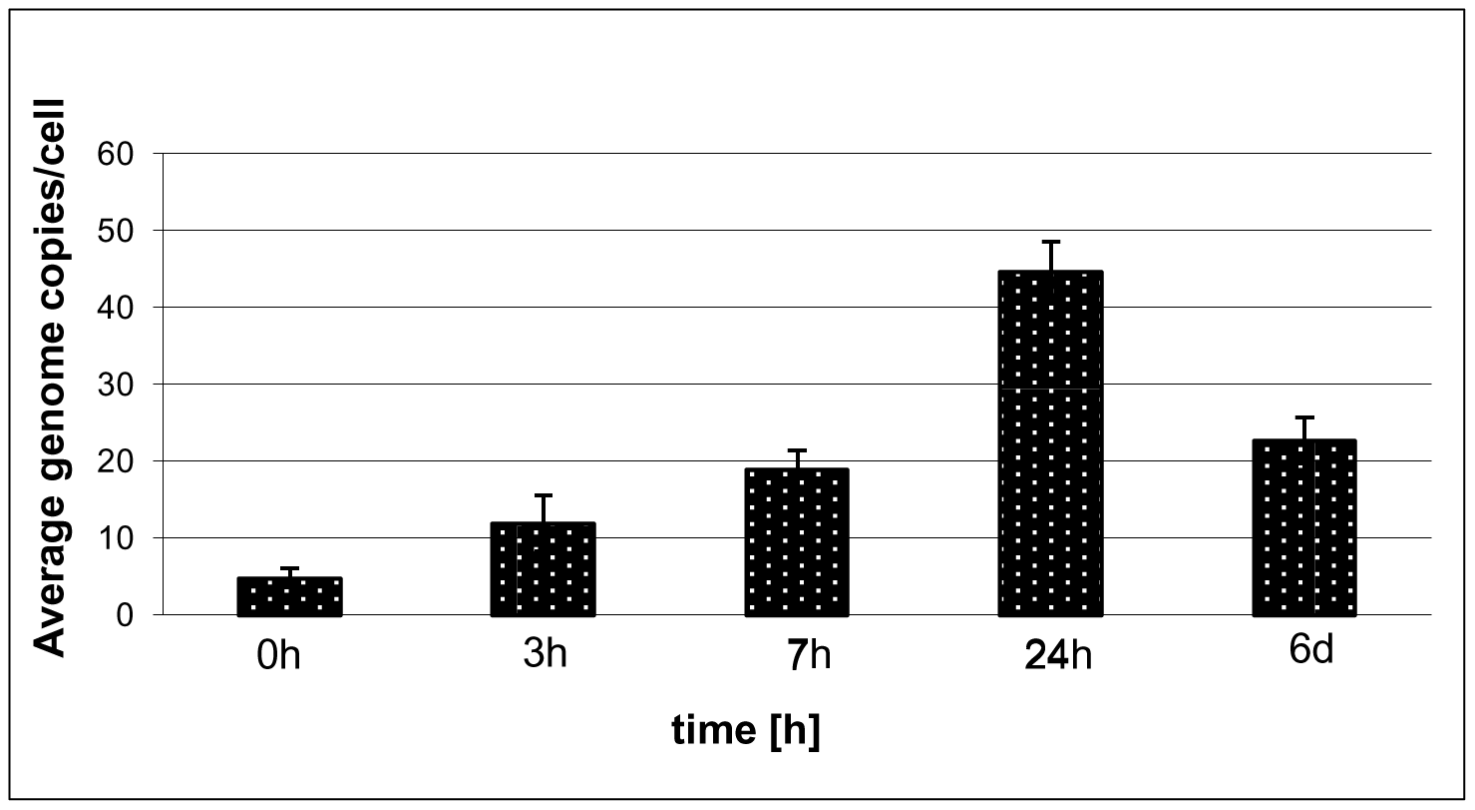
Chromosome copy numbers after re-addition of phosphate to starved cells. Stationary phase, phosphate-starved, chromosome-depleted cells were resuspended in medium containing 1 mM phosphate. At various times, as indicated, aliquots were removed and the chromosome copy number was determined using Real Time PCR. Three biological replicates were performed and average values and standard deviations are shown.

Polyploidy dependence upon nutrient availability was further substantiated when cells from P-starved stationary phase cultures that were depleted of extra chromosomes were amended with phosphate: within three hours the chromosome copy number more than tripled and within 24 hours they increased by greater than 10-fold to more than 40 copies per cell (Fig. 5). Thus phosphate-starved *Hfx. volcanii* cells take up phosphate very fast after re-addition and use it to re-establish the polyploid state, even with an overshoot phase with more than 40 chromosomal copies per cell.

The genome sequence of *Hfx. volcanii* contains five genes that are annotated to encode polyphosphate kinases (HVO_0074, HVO_0837, HVO_1650, HVO_2363, HVO_2598), opening the possibility that *Hfx. volcanii* might also use polyphosphate as a phosphate-storage polymer, in addition to genomic DNA. To investigate this possibility, *Hfx. volcanii* was grown in the presence of added phosphate and a culture aliquot was removed during exponential growth. The cells were fixed and stained with DAPI to simultaneously detect genomic DNA as well as polyphosphate based on the differential wavelengths of fluorescence emission for these biopolymers [18]. Chromosomal DNA was readily observed in cells using this approach, in contrast to polyphosphate, which was not detected (data not shown). Therefore, at least under the conditions of the experiments of this study, *Hfx. volcanii* appears to use only genomic DNA as polymer for the storage of phosphate and not polyphosphate. However, it should be noted that even the detection of polyphosphate would not have disproven our observation that *Hfx. volcanii* uses genomic DNA as a phosphate storage polymer.

### During phosphate starvation other phosphate containing biomolecules are produced from genomic DNA, not from rRNA

The results showed that during growth under phosphate starvation *Hfx. volcanii* dramatically decreased its chromosome copy number from about 30 to only 2, suggesting that it uses genomic DNA as a phosphate storage polymer. Another possible source of phosphate might be ribosomal RNA. The numbers of ribosomes per cell are influenced by parameters like growth rate and it can vary widely, both in *E. coli* [19] and in *Hfx. volcanii* [20]. Therefore, for a better understanding of the phosphate balance of cells during phosphate starvation, also the number of ribosomes was quantified.

*Hfx. volcanii* cultures were again grown in the absence of added P. The cell density was quantified and increased from 3.22×10^7^ cells ml^−1^ to 2.70×10^8^ cells ml^−1^. This is an 8.4-fold increase in cell number, which is in excellent agreement with the 8.5-fold increase in OD_600_ observed in previous experiments (compare Fig. 3). The number of ribosomes prior to and after phosphate starvation was quantified using a previously described approach [20]. Cells of the preculture grown in complex medium contained 29250 ribosomes (SD 1290, n = 3), a number similar to the number of 26000 ribosomes per cell determined earlier [20]. Stationary phase cells after phosphate starvation contained on average 3290 ribosomes (SD 97, n = 3). This is an 8.8-fold reduction of the number of ribosomes per cell during phosphate starvation, a value that is very similar to the 8.4-fold increase in cell number during phosphate starvation. Together these results revealed that ribosomal RNA is neither source nor sink of phosphate during phosphate starvation, but that ribosomes are distributed among the daughter cells and that the phosphate content bound in rRNA is self-sufficient during phosphate starvation.

*Hfx. volcanii* harbors not only the major chromosome, but also three additional small chromosomes and a very small plasmid. To enable a comprehensive comparison of the total amount of phosphate bound in rRNA and in DNA, three independent cultures were again grown in the absence of added phosphate and the numbers of four replicons were quantified prior to and after phosphate starvation (the replicon pHV2 is a very small plasmid that is not present in strain H26). The results are summarized in Table 1. As expected, the numbers of all replicons were severely reduced after phosphate starvation. It was revealed that a polyploid *Haloferax* cell growing exponentially in complex medium contains about 2.2×10^8^ molecules of phosphate in its DNA. With approximately 4600 nucleotides per ribosome and 29250 ribosomes per cell, the estimated total amount of phosphate in rRNA is 1.2×10^8^ molecules per cell. Thus in the polyploid *Hfx. volcanii* the amount of phosphate bound in DNA is about twice that bound in ribosomes. This is contrast to monoploid species, which contain more phosphate in rRNA than in genomic DNA.

**Table 1:** Phosphate content of the four *Hfx. volcanii* chromosomes before and after growth in the absence of phosphate.

Increase in cell No.		1×		8.4×	
Replicon	Size [bp]	Ploidy before.	P atoms	Ploidy after	P atoms
**Chromosome**	2.85×10^6^	30	1.7×10^8^	2	9.6×10^7^
**pHV1**	8.51×10^4^	21	3.5×10^6^	2	2.9×10^6^
**pHV3**	4.38×10^5^	26	2.3×10^7^	4	2.9×10^7^
**pHV4**	6.36×10^5^	17	2.1×10^7^	2	2.1×10^7^
**sum [bp]**	**4.0×10^6^**	**sum P atoms**	**2.2×10^8^**		**1.5×10^8^**

**Figure 6:**
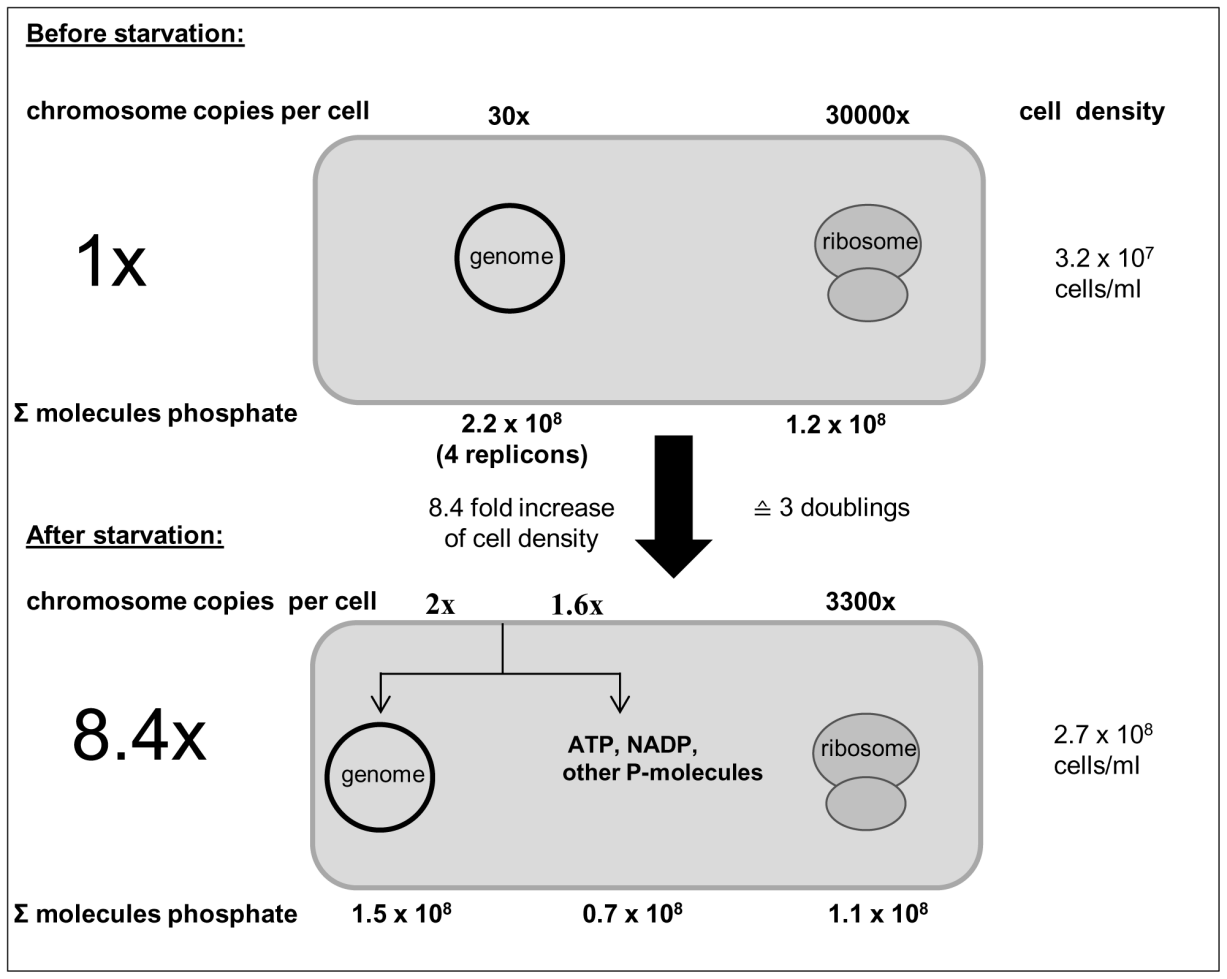
Phosphate balance in cells prior to and after growth in the absence of external phosphate. A preculture in complex medium was grown to mid-exponential phase. Aliquots were harvested, washed, and used to inoculate synthetic medium lacking any added phosphate source. Aliquots were removed at the beginning of the experiment and after growth in the absence of phosphate ceased. The cell densities were quantified using a counting chamber, the genome copy numbers were quantified by Real Time PCR, and the numbers of ribosomes were quantified after RNA isolation and two DNase treatments as described in the text. The figure gives a schematic overview of the phosphate balance prior to and after growth during phosphate starvation.

Fig. 6 summarizes the balancing of phosphate during growth of *Hfx. volcanii* under phosphate starvation, which is based on the quantification of the numbers of cells, chromosomes and ribosomes. Taken together, the results revealed that the numbers of rRNA-bound phosphate molecules are identical prior to and after phosphate starvation, and thus ribosomal RNA is neither source nor sink of phosphate. In contrast, only about 2/3 of the phosphate that was DNA-bound prior to phosphate starvation was still found in chromosomes after starvation. This indicates that 1/3 of the chromosomes had been degraded and suggests that they were the source of intracellular phosphate for the production of other phosphate-containing biomolecules, e.g. phospholipids, phosphoproteins, phosphosugars, ATP, NADP^+^, etc. Thus it seems that the polyploid *Hfx. volcanii* uses chromosomal DNA as a phosphate storage polymer in two different ways: 1) cell division in the absence of replication is enabled by distribution of preexisting chromosomes to the daughter cells, and 2) chromosomal DNA is degraded to liberate phosphate needed for other biomolecules that do not have a storage pool in the cell.

### Growth during phosphate starvation diminishes genetic advantages of polyploidy

**Figure 7:**
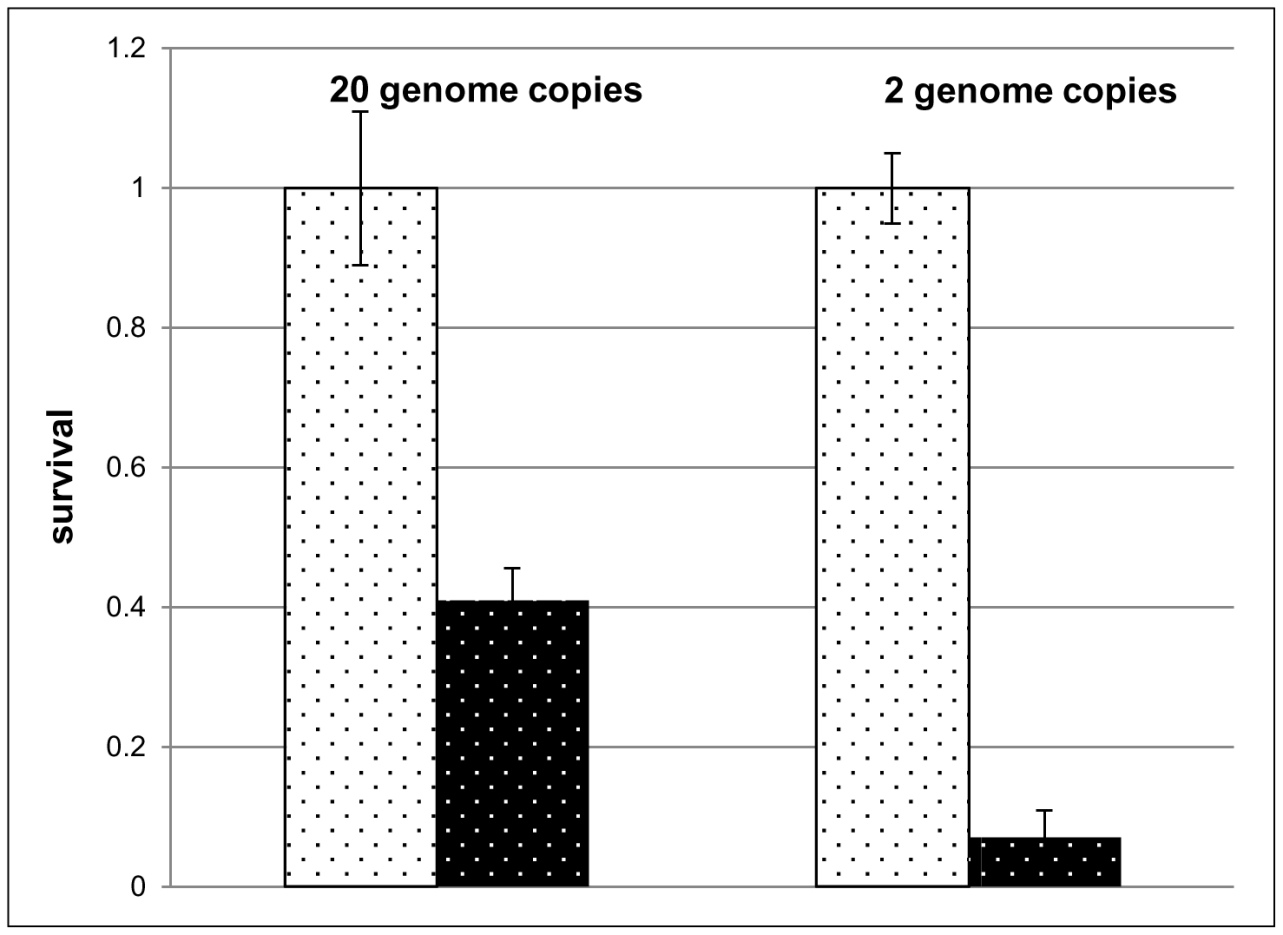
Desiccation resistances of cells of with different ploidy levels. Cultures were grown to mid-exponential phase in synthetic medium with casamino acids and 1 mM K_2_HPO_4_ to generate cells with 20 copies of the chromosome. For comparison, cultures were grown in synthetic medium in the absence of phosphate to generate cells with 2 copies of the chromosome. Both types of cells were exposed to a desiccation period of 12 days. Colony forming units (CFU) were quantified before and after desiccation, and the survival rates were calculated. Average results of three independent experiments and their standard deviations are shown. Left columns, prior to desiccation, right columns, after desiccation.

Various genetic advantages of polyploidy for haloarchaea have been proposed [21]. The severe reduction of the ploidy level during growth under phosphate starvation indicates that *Hfx. volcanii* might diminish genetic advantages in favor of cell density increase. To experimentally test this hypothesis, the desiccation resistances of cultures with different chromosome copy numbers were quantified. The desiccation resistance of two types of cultures was analyzed, 1) cultures grown in synthetic medium with casamino acids and 1 mM phosphate to exponential phase, which contained 20 copies of the chromosome, and 2) cultures grown in the absence of phosphate, which contained 2 copies of the chromosome. Both types of cultures were exposed to a 12 day desiccation period. Prior to and after desiccation, the numbers of colony forming units were quantified, and the survival rates were calculated. Fig. 7 shows that 40% of the polyploid cells survived desiccation, while only 8% of the diploid cells survived desiccation. Thus the reduction of the ploidy level from 20 to 2 was accompanied by a fivefold reduction in desiccation resistance. These results show that during phosphate starvation *Hfx. volcanii* diminishes at least one genetic advantage of polyploidy and instead drastically reduces the copy number to enable sustained growth in the absence of external phosphate.

## Discussion

### Environmental genomic DNA as a nutrient source

DNA is an information storage polymer for all cells, yet for some species of prokaryotes this molecule also has a nutritional role. We could show that *Hfx. volcanii* can grow on external (environmental) genomic DNA as a sole source of carbon, nitrogen, or phosphate. This has also been observed for other species, e.g. the bacterium *Shewanella* [22] as well as for the cyanobacterium *Synechocystis* sp. strain PCC6803 (Zerulla and Soppa, unpublished data). Various additional bacteria can use DNA as a nutrient source [23]. Enrichment cultures with DNA as the sole source of C, N, P, and energy have been successful and resulted in the isolation of assemblages of diverse bacteria [24].

DNA is present in a variety of environments around the world with concentration from <1 µg/l to more than 70 µg/l [25]–[27]. Nitrogen and phosphorus are limiting nutrients in a variety of ecosystems and significant amounts of dissolved environmental phosphorus is often locked in DNA indicating that it is an excellent extracellular source of phosphorus [28].

### Genomic DNA as an intracellular phosphate storage polymer

Many evolutionary advantages of polyploidy exist, and for haloarchaea alone nine different evolutionary advantages have recently been proposed, including low mutation rate, high resistance to desiccation, gene redundancy, survival over a geological time scale and shifting gene regulation from stochastics to statistics [21]. Here we add an additional evolutionary advantage of polyploidy, i.e. the usage of genomic DNA as a storage polymer for phosphate. The results suggest that *Hfx. volcanii* uses genomic DNA as a phosphate storage polymer under phosphate starvation in two different ways, 1) the high copy number of chromosomes enables an eightfold increase in cell density, which is equivalent to three doublings, in the absence of replication, and 2) about 1/3 of the chromosomes are degraded to liberate phosphate needed for the synthesis of other phosphate containing biomolecules (see Fig. 6). It should be noted that the latter result is indirect and was calculated from the numbers of cells and chromosomes prior to and after phosphate starvation. If the preexisting chromosomes would only be distributed to the daughter cells and no DNA degradation would take place, the 8.4-fold increase in cell number would lead to an average copy number of 3.6 chromosomes per cell (Fig. 4). The experimentally determined real number of chromosomes per cell after starvation was 2.0 (very low standard deviation, see Fig. 4). To test the statistical significance of the difference between these two values, an unpaired t-test was performed. As three biological replicates were measured and for each replicate four different dilutions were quantified in duplicates, each value rests on 24 technical replicates. The P value was found to be 1.8×10^−11^ and thus the difference between these two values is highly significant, strongly suggesting that indeed about 1/3 of the chromosomes were degraded to liberate phosphate for other phosphate containing biomolecules.

Notably, this was not the case for phosphate bound in ribosomes. The number of ribosomes per cell is severely decreased during growth under phosphate starvation (from 30000 to 3300). However, ribosomes are only distributed to the descendent cells and the sum of rRNA-bound phosphate molecules stays constant. Therefore, during growth under phosphate starvation ribosomes are neither a source nor a sink of phosphate, in contrast to genomic DNA. It should be noted that in polyploid species the fraction of phosphate bound in DNA is higher than that in RNA (see Fig. 6). In contrast, in monoploid species more phosphate is bound in RNA than in DNA. For example, *E. coli* cells growing with a generation time of 24 minutes contain 3.9×10^8^ molecules of phosphate bound to RNA, but only 3.0×10^7^ molecules of phosphate bound to DNA [19].

Chromosomes and ribosomes have high copy numbers that can be severely reduced during growth under phosphate starvation. This does not seem to be possible for other phosphate-containing biomolecules, e.g. phospho-lipids or ATP. The membrane of haloarchaea contain a high fraction of phospholipids [29], [30], therefore, the 8.4-fold increase in cell number observed during phosphate starvation depends on extensive phospho-lipid synthesis. Similarly, it seems unlikely that the cells can grow with 8.4-fold reduced levels of ATP, NADP^+^, etc. Therefore, the cells must have an internal phosphate storage pool to explain the observed growth in the absence of external phosphate. As phosphate was not liberated from ribosomes, polyphosphate was not detected, and no other phosphate-storage polymer is known in haloarchaea, this is another indication that genomic DNA was indeed used as a phosphate storage polymer that was partially degraded to liberate phosphate.

Recently it became clear that polyploid prokaryotes are no seldom exceptions, but that polyploidy is widespread in archaea and bacteria [4]–[9], [31]. Therefore, it might well be that the usage of genomic DNA as intracellular nutrient storage polymer is not limited to *Hfx. volcanii*, but is present in many additional species. The analysis of selected additional polyploid species is currently under way.

That DNA has roles other than storing genetic information is exemplified by its stabilizing function in biofilms. Examples have been reported that DNA is essential for biofilm formation and that mutants unable to export DNA lose the ability to form biofilms [32]. Also haloarchaea including *Hbt. salinarum* and *Hfx. volcanii* have been shown to export DNA when they form biofilms [33]. Not only nature makes use of DNA that exceeds its role as genetic material, the many applications of “DNA origami” [34] demonstrate that DNA is an organic polymer that might have a bright future in bionanotechnology.

### Growth during phosphate starvation diminishes genetic advantages of polyploidy

During phosphate starvation *Hfx. volcanii* dramatically decreases the number of chromosomes and in fact gives up polyploidy. This led to the prediction that the various genetic advantages of polyploidy should be lost at the end of growth in the absence of phosphate. This prediction was tested using one evolutionary advantage of polyploidy, i.e. the resistance to desiccation. It could indeed be shown that phosphate-starved cells with two chromosomes are five-fold more sensitive to desiccation than cells with the normal number of about 20 chromosomes (compare Fig. 7), and thus it could be experimentally verified that *Hfx. volcanii* diminishes at least one genetic advantage of polyploidy upon phosphate starvation. It will be interesting to investigate whether also other species prefer to grow under phosphate starvation and give up genetic advantages of polyploidy, or whether different strategies exist.

### An alternative explanation for the evolutionary origin of DNA: a hypothesis

The distribution of polyploidy in archaea and bacteria indicates that polyploidy has evolved independently at different times for different reasons in various phylogenetic groups, and thus recent species would have developed polyploidy rather late in evolution. The universal conversation of RecA (RadA/Rad51) in all life forms indicates that homologous recombination is very ancient, and this might be also true for the initial development of all advantages of polyploidy that require homologous recombination. Nevertheless, polyploidy might even predate the invention of homologous recombination. Thus, we suggest that the evolutionary origin of polyploidy was initiated by a need to store phosphate intracellularly in a safe and readily utilizable form. In essence, we propose that the usage of DNA as a phosphate storage polymer might by far predate all other evolutionary advantages of polyploidy and that in fact the first “polyploid” cell had many “genome” copies without using DNA as genetic material.

Many controversial theories about the origin of life and the evolution to a free-living modern-type of cell exist, ranging from pyrite catalyzed early metabolism to a *bona fide* “RNA world”. However, all of these theories agree that RNA by far predates DNA as the molecule encoding heritability. The current concept is that DNA evolved because its higher stability compared to RNA made it the material of choice to store genetic information, and DNA genomes replaced the previous RNA genomes. Given our evidence for DNA's dual nature as a molecule for information and phosphorus storage, we propose the parsimonious argument that the development of DNA might have stemmed from the need to store phosphate intracellularly, and then added the role of genetic storage once mechanisms for template-based replication and transcription were invented. The driving force for the early development of DNA as storage polymer would have been its much greater stability in comparison to an alternative phosphate storage polymer, e.g., polyphosphate. Hydrolysis of polyphosphate is exergonic, and its stability is highly influenced by pH and temperature [35]. While at neutral pH and room temperature polyphosphate is reasonably stable and complete hydrolysis requires several months, at acidic pH or elevated temperatures it occurs within hours [35]. Notably, even at neutral pH and room temperature DNA is much more stable than polyphosphate. Of course the proposal that the functionality as a storage polymer might have been the driving force in the development and acquisition of DNA during the early evolution of life is nothing else than a hypothesis. While it might not be true and cannot be proven, it opens an alternative view on possible developments in the pre-DNA world.

## Methods

### Haloarchaeal strain and media

*Haloferax volcanii* strain H26 was kindly provided by Thorsten Allers (University of Nottingham, UK). It was and grown in complex medium [36] or in synthetic medium [37] supplemented with 8 µM FeSO_4_ (Roth, P015.1), 0,1% (v/v) SL-6 trace element solution [38] (all from Roth), 1 ml vitamin solution (Sigma Aldrich, B6891), 50 µg ml^−1^ uracil (Applichem, A0667) and 100 mM MOPS pH 7.2 (Sigma Aldrich, M3183). All components of the synthetic medium were of the grade “per analysis” and thus free of phosphate, e.g. K_2_HPO_4_ (Roth, 6878.2), NH_4_Cl (Applichem, A0988), glucose (Merck, 1083441000), NaCl (Roth, 3957.5), MgCl_2_ (Roth, 2189.1), MgSO_4_ (Applichem, A1037), KCl (Roth, 6781,1), CaCl_2_ (Applichem, A3587), and Tris (A1086). If not otherwise stated, the synthetic medium was also supplemented with 0.5% (w/v) glucose as a C source, 10 mM NH_4_Cl as a N source, and 1 mM K_2_HPO_4_ as a P source. For growth experiments with DNA as a source of P K_2_HPO_4_ was omitted and genomic DNA was added to a final concentration of 250 µg/ml. Cultures were grown in Erlenmeyer flasks in a rotary shaker at 42 °C and 250 rpm or in microtiter plates as described below.

### Growth in microtiter plates

For growth studies in microtiter plates a preculture was grown in an Erlenmeyer flask in complex medium to exponential phase (OD_600_ 0.4), washed three times in basal salts (medium without a carbon, nitrogen and phosphate source), and resuspended in medium specific to the experiment. The OD_600_ was determined and the cultures were diluted, so that the start OD_600_ in the 150 µl culture volume in the microtiter plate was about 0.025 (which is equivalent to 0.5 using normal cuvettes with a pathlength of 1 cm). Microtiter plates were incubated in an orbital shaker (Heidolph, Schwalbach, Germany) at 42 °C with a shaking velocity of 1100 r.p.m. The OD_600_ values of triplicate cultures was measured at the time points indicated in the respective figures using the microtiter plate photometer Spectramax 340 (Molecular Devices, Ismaning, Germany). Average values of three independent cultures and their standard deviations were calculated.

### DNA extraction and purification

Chromosomal DNA isolated from *Hfx. volcanii* H26 cells by spooling and ethanol precipitation (www.haloarchaea.com/resources/halohandbook). DNA was dissolved in 10 mM Tris-Cl solution (pH 8, in DNA-grade water). Purified DNA was used fresh for growth experiments to avoid any subsequent hydrolysis. DNA concentrations were determined prior to supplementation spectrophotometrically using a Nanodrop ND-1000. DNA samples were visualized on agarose gels prior to supplementation and found to be of high molecular weight (data not shown).

### Verification of the usage of high molecular weight chromosomal DNA

*Hfx. volcanii* was grown in Erlenmeyer flasks in synthetic medium with casamino acids (0.25% (w/v)) as carbon and energy source in the presence of 100 µg/ml genomic DNA as phosphate source. The culture was started with an OD_600_ of 0.1 and grown into stationary phase. At various time points 1 ml aliquots were removed and cells were removed by centrifugation (8000 g, 5 min, room temperature). 30 µl aliquots of the supernatants were dialyzed on membrane filters (Millipore, 13 mm diameter, VSWP01300) against distilled water and analyzed by analytical agarose gel electrophoresis.

### Quantification of ploidy levels using quantitative real-time PCR

Precultures were grown in complex medium to a cell density of about 2×10^8^ cells/ml. Aliquots of 3×10^8^ cells were removed and the cells were harvested by centrifugation (4000 rpm, 30 min) and washed in basal salt solution (medium without carbon and phosphate source). The cells were used to inoculate synthetic medium with glucose as carbon source and 10 mM NH_4_Cl as nitrogen source. K_2_HPO_4_ was added as a phosphate source at concentrations described in the respective experiments, or phosphate was omitted. Samples for the quantification of the replicon copy number were collected during exponential phase (without added phosphate: 9.4×10^7^ cells/ml; with 1 or 10 mM phosphate: 5.2×10^8^ cells/ml) and at stationary phase (without added phosphate: 2.7×10^8^ cells/ml; with 1 or 10 mM phosphate: 1.3×10^9^ cells/ml). A RT-PCR approach was applied for the determination of chromosome copy numbers as described previously [4]. Standard fragments of about 1 kbp were amplified by PCR using total DNA of *Hfx. volcanii* as template (oligonucleotides see Table S1). Purification of the standard fragment and preparation of the cell extracts were essentially performed as described [38]. 3×10^8^ cells were collected by centrifugation, resuspended in 100 µl basal salt solution, lysed by the addition of 900 µl water, and dialyzed on membrane filters (Millipore, 13 mm diameter, VSWP01300) against distilled water. RT-PCR conditions were 10 min 96°C, 40 cycles with 30 s 96°C, 30 s 62°C, 30 s 72°C followed by 5 min 72°C and a melt curve analysis from 62°C to 96°C in 1°C steps. In each case three independent cultures (biological replicates) were analyzed. For each replicate four different dilutions of the extracts were measured in duplicates, so that 24 technical replicates were used to calculate the average copy numbers and their standard deviations. The standard curve was comprised of serial tenfold dilutions of the standard fragment (10^3^-fold to 10^8^ fold) that were measured in duplicates. It was ensured that all PCR reactions were exponential, i.e. the C_T_ differences of tenfold dilutions were about 3.3. A negative control with the omission of template DNA was also performed.

### Statistical analysis

The experimentally determined average number or genome copies after growth in the absence of phosphate was 2. The expected average value if the chromosomes present in the inoculum would have been distributed to the daughter cells and no degradation would have occurred was 3.6. To unravel whether these two values are significantly different an unpaired t-test was performed using Excel and the respective results of the technical replicates of the three biological replicates.

### Analysis of potential polyphosphate formation

*Hfx. volcanii* was grown in synthetic medium with casamino acids as carbon and energy source in the presence of 1 mM phosphate to mid-exponential growth phase. Cells were harvested by centrifugation and fixed with formaldehyde as described previously [39]. They were stained with DAPI (500 µg/ml) for 10 minutes at room temperature as described by [18] and analyzed by fluorescence microscopy.

### Quantification of the number of ribosomes

The number of ribosomes was quantified as described by [20]. In short, total RNA was isolated and residual DNA was removed by two consecutive treatments with RNase-free DNase according to the instructions of the supplier (Qiagen, 79254). Each time the DNase treatment was followed by an ethanol precipitation [40]. The RNA concentration was determined spectroscopically and the fraction of ribosomal RNA and the molecular weight of ribosomal RNA was used to calculate the number of ribosomes per cell [20].

### Quantification of desiccation survival

*Haloferax volcanii* cultures were grown in synthetic medium with casamino acids and with 1 mM K_2_HPO_4_ to mid-exponential phase or without K_2_HPO_4_ to stationary phase. Subsequently the cells were washed in basal salt solution and were concentrated 20-fold in basal salt solution. Thereafter the cells were placed onto glass microscope cover slips (2×50 µl per cover slip as 4–5 dots) and allowed to dry completely. For desiccation at ambient pressure, cover slips were placed in an exsiccator with Drierite desiccant (97% CaSO_4_ and 3% CoCl_2_) at 37°C for 12 days. Cells were rehydrated by placing cover slips in 5 ml basal salt solution with moderate agitation for 30 minutes at room temperature. The resulting cell suspensions were concentrated by centrifugation with 5000×g at room temperature for 10 min, resuspended in 1 ml basal salt solution. Serial dilutions were prepared and triplicate aliquots were plated onto solid complex medium to quantify cell survival. Colony forming units were quantified after 4–7 days of incubation at 42°C. Control samples were processed in the same manner immediately after preparation and were not permitted to dry.

## Supporting Information

Table S1 **Oligonucleotides used for quantification of the copy numbers of four replicons using a Real Time PCR method.**
(DOCX)
