# Supplementary material for: DNA as a Phosphate Storage Polymer and the Alternative Advantages of Polyploidy for Growth or Survival
Source: PLoS One. 2014 Apr 14;9(4):e94819. doi: 10.1371/journal.pone.0094819 (PMC3986227; doi:10.1371/journal.pone.0094819)
Supplement: Table S1 — Oligonucleotides used for quantification of the copy numbers of four replicons using a Real Time PCR method. (DOCX) [file pone.0094819.s001.docx]

Table S1: Oligonucleotides used for replicon copy number determination

| Primer | 5´-3´ | Application |
| --- | --- | --- |
| leuB_neu-L1 | GATATCAAGGCCGTCGGCGACTTCGAG | standard fragment |
| leuB_neu_R2 | GATATCCAGAGTCGGTCGAGAATC | standard fragment |
| RT_A_leuB-wt_for4 | GCGAGACGGACGGTCGCTTCC | qPCR |
| RT_A_leuB-wt_rev4 | CGAGCAGCATCGCCGCGGACAG | qPCR |
| pHV4_St_for1 | GGACACCGAGATCTATCGGGACCGGGACGC | standard fragment |
| pHV4_St_rev1 | GGCGAGTTCGGTAAACCGGGTGTAGCGGGG | standard fragment |
| pHV4_A_for1 | GCATTCTCTACCAACTTCCGCGAGCGCGGG | qPCR |
| pHV4_A_rev1 | GCTCTTCTTCGATGCGGTCGGTGTCGTCGTCG | qPCR |
| pHV3_St_for1 | CGCGACCCGCTGTTCCGCTATGACCAACC | standard fragment |
| pHV3_St_rev1 | GGTCGTCGGCGACGGTGAGATACTGTCGG | standard fragment |
| pHV3_A_for1 | GCACACCGAGGCCTCGGTCATCAAGACGC | qPCR |
| pHV3_A_rev1 | GCGTCGTACGACGGGAAGACGAGTTCGTCG | qPCR |
| pHV1_St_for1 | CGGGAGATACCCGAGACGACGAGATGCAACAGC | standard fragment |
| pHV1_St_rev1 | GCGATTTTATCCCGGTCCACGTCAGACCGAAGC | standard fragment |
| pHV1_A_for1 | CCAGCACAATACGGGGTATCAGCTCACCCGC | qPCR |
| pHV1_A_rev1 | GGTTCGAGGTGTAAGCGTCGAACTTCACCGTCC | qPCR |
